# Genetic diversity of stilbene metabolism in *Vitis sylvestris*

**DOI:** 10.1093/jxb/erv137

**Published:** 2015-04-06

**Authors:** Dong Duan, David Halter, Raymonde Baltenweck, Christine Tisch, Viktoria Tröster, Andreas Kortekamp, Philippe Hugueney, Peter Nick

**Affiliations:** ^1^Molecular Cell Biology, Botanical Institute 1, Karlsruhe Institute of Technology, Kaiserstr. 2, 78133 Karlsruhe, Germany; ^2^Métabolisme Secondaire de la Vigne, UMR 1131, INRA, Université de Strasbourg, 28 rue de Herrlisheim, F-68021 Colmar, France; ^3^DLR Rheinpfalz State Education and Research Center of Viticulture and Horticulture and Rural Development, Breitenweg 71, D-67435 Neustadt, Germany

**Keywords:** Basal immunity, breeding, defence, genetic diversity, grapevine (*V. sylvestris*), stilbenes, UV-C.

## Abstract

Stilbenes, as important secondary metabolites of grapevine, represent central phytoalexins and therefore constitute an important element of basal immunity. In this study, potential genetic variation in *Vitis vinifera* ssp. *sylvestris*, the ancestor of cultivated grapevine, was sought with respect to their output of stilbenes and potential use for resistance breeding. Considerable variation in stilbene inducibility was identified in *V. vinifera* ssp. *sylvestris*. Genotypic differences in abundance and profiles of stilbenes that are induced in response to a UV-C pulse are shown. Two clusters of stilbene ‘chemovars’ emerged: one cluster showed quick and strong accumulation of stilbenes, almost exclusively in the form of non-glycosylated resveratrol and viniferin, while the second cluster accumulated fewer stilbenes and relatively high proportions of piceatannol and the glycosylated piceid. For all 86 genotypes, a time dependence of the stilbene pattern was observed: piceid, resveratrol, and piceatannol accumulated earlier, whereas the viniferins were found later. It was further observed that the genotypic differences in stilbene accumulation were preceded by differential accumulation of the transcripts for chalcone synthase (*CHS*) and stilbene-related genes: phenylalanine ammonium lyase (*PAL*), stilbene synthase (*StSy*), and resveratrol synthase (*RS*). A screen of the population with respect to susceptibility to downy mildew of grapevine (*Plasmopara viticola*) revealed considerable variability. The subpopulation of genotypes with high stilbene inducibility was significantly less susceptible as compared with low-stilbene genotypes, and for representative genotypes it could be shown that the inducibility of stilbene synthase by UV correlated with the inducibility by the pathogen.

## Highlight

We show that the ancestor of cultivated grapevine harbours genetic factors that increase the inducibility of stilbenes correlated with increased resistance to the important pathogen grapevine downy mildew.

## Introduction

Stilbenes are a small family of plant secondary metabolites derived from the phenylpropanoid pathway, which are found in a limited number of plant species (Langcake and Pryce, 1976; Kodan *et al.*, 2001; Yu *et al.*, 2005). In the *Vitaceae*, stilbenes are important phytoalexins, which accumulate in response to various biotic and abiotic stresses such as pathogen attack (Langcake and Pryce, 1976; Adrian *et al.*, 1997; Schnee *et al.*, 2008), UV-C irradiation (Bais *et al.*, 2000), application of chemicals such as aluminium ions and ozone (Rosemann *et al.*, 1991; Adrian *et al.*, 1996), or salinity stress (Ismail *et al.*, 2012). They can also be induced in response to plant hormones, such as jasmonates and ethylene (Belhadj *et al.*, 2008a, b; D’Onofrio *et al.*, 2009). In grapevine, the stilbene *tran*s-resveratrol (*trans*-3,5,4’,-trihydroxy-*trans*-stilbene) has attracted particular attention, not only because of its antimicrobial activity, but also due to its possible pharmacological benefits to humans. The relatively low incidence of coronary disease in France despite a diet rich in saturated fatty acids (popularized as the ‘French Paradox’) has been attributed to regular intake of resveratrol associated with moderate consumption of red wine (Siemann and Creasy, 1992). Accumulating evidence indicates that this natural product can prevent some diseases, such as cardiovascular diseases, cancers, obesity, diabetes, and neurodegenerative diseases, and in addition can cause an extension of life span (for reviews, see Baur and Sinclair, 2006; Roupe *et al.*, 2006).

In a previous work, it was shown for cell cultures from *Vitis rupestris* and *V. vinifera* cv. ‘Pinot Noir’ that stilbene patterns differ depending on the genotype (Qiao *et al.*, 2010; Chang *et al.*, 2011; Chang and Nick, 2012). Cell lines derived from two distinct genotypes showed different responses to elicitation with flg22 or Harpin. Whereas most of the early defence responses overlapped in both cell lines, they differed in the induction of pathogenesis-related (PR) genes, synthesis and metabolism of stilbene phytoalexins, and the execution of hypersensitive response (HR)-mediated cell death. In the resistant *V. rupestris*, resveratrol was oxidized to toxic δ-viniferin, whereas in the susceptible cv. ‘Pinot Noir’, it was preferentially accumulated in form of its non-toxic glucoside piceid. This suggests that there is genetic variation within the genus *Vitis* with respect to stilbene profiles and, since bioactive stilbenes such as resveratrol or δ-viniferin harbour antimicrobial activity, this genetic variation might be exploited for sustainable viticulture.

Crop wild relatives (CWRs) have shifted into the centre of the attention of plant breeding and evolutionary biology (Ellstrand *et al.*, 2010), because they represent valuable genetic resources for breeding. The cultivated grape *V. vinifera* L. ssp. *vinifera* has played an important role with respect to economy and culture over many centuries. It represents one of the most important crops worldwide considering its global distribution and its high economic value. However, its ancestor and CWR species, the European wild grape *V. vinifera* L. ssp. *sylvestris* Hegi, is close to extinction. In the frame of a project designed to conserve this species *ex situ*, an extensive collection of the European wild grape (for simplicity termed *V. sylvestris*) representing a complete copy of the genetic variation still present in Germany has been established (Nick, 2012). A closer analysis of this collection revealed that many genotypes show good tolerance against several grapevine diseases, such as downy mildew (*Plasmopara viticola*), powdery mildew (*Erysiphe necator*), and black rot (*Guignardia bidwelli*), which were all introduced only 150 years ago from North America (Tisch *et al.*, 2014). Plant immunity is made up of two levels: an evolutionarily ancient basal immunity is complemented by a more efficient and specific second line of defence. This specific immunity has evolved during a long arms race between pathogen and host plant. Since cultivated grapevine (*V. vinifera* ssp. *vinifera*) did not evolve together with these recently introduced pathogens, it represents a naive host and, in contrast to North American wild species of *Vitis*, lacks the efficient second layer of innate immunity against these diseases. The fact that some genotypes of *V. sylvestris* can withstand these diseases is likely to be due to a more efficient basal immunity.

Since phytoalexins, such as the stilbenes, represent a central element of basal immunity, the aim of this work is to characterize the diversity of this *V. sylvestris* collection with respect to its capacity for stilbene biosynthesis, which might be exploited as a genetic resource for resistance breeding. *Vitis sylvestris* was therefore screened as the ancestral species for genotypic differences in stilbene accumulation (stilbene ‘chemovars’). Since the response to pathogens is subject to considerable variation and dependent on seasonal influences, a short pulse of UV-C light was used as a well controllable trigger. Using this approach, it is shown in the current study that there is, in fact, considerable genetic variation in *V. sylvestris* concerning stilbene output. A few *V. vinifera* cultivars were included for reference. It is confirmed that different stilbene patterns exist not only in cell lines, but also in the ‘real world’. In addition, *V. sylvestris* chemovars that produce high levels of the bioactive viniferins are identified and it is shown that these chemovars are less susceptible to infection by downy mildew of grapevine (*P. viticola*).

## Materials and methods

### Plant material

The *Vitis vinifera* ssp. *sylvestris* plants used in this study were collected (as cuttings) from natural sites at the ‘Ketsch’ peninsula at the Rhine River, in Southern Germany, which harbours the largest natural population in Central Europe (these accessions are indicated by ‘Ke’). Additionally, 25 *V. sylvestris* individuals originating from different sites in the Upper Rhine Valley (from the Hördt peninsula, indicated by ‘Hoe’) were included in this study; details of the collection sites have been described (Ledesma-Krist *et al.*, 2014). Also included were six *V. vinifera* cultivars common in German and French vineyards (Augster Weiss, Pinot Blanc, Pinot Noir, Müller-Thurgau, Chardonnay, and Cabernet Sauvignon), along with one American (*V. rupestris*), and one Chinese (*V. quinquangularis*) species. All accessions are maintained as living specimens in the grapevine collection of the Botanical Garden of the Karlsruhe Institute of Technology, and have been photographically documented, and re-determined using morphological keys and ampelographic descriptors of the Organisation Internationale de la Vigne et du Vin (Olmo, 1976). For stilbene analysis, leaves of vineyard-grown plants were used over two subsequent years (2012 and 2013). For RNA extraction, the leaves were harvested from greenhouse-grown plants cultivated at a temperature of 22 °C and 18 °C (day and night, respectively) and a photoperiod of 14h light and 10h dark.

### Preparation of leaf samples

To obtain fully expanded leaves of uniform size and comparable developmental state, the fourth and fifth leaves, counted from the apex, were excised from randomly selected individuals of the respective genotype, subjected to UV-C stress as described below, and incubated upside down on moist filter paper in large Petri dishes. For the UV-C treatment, the abaxial surface of the entire leaf was exposed to UV-C light (254nm, 15W, Germicidal, General Electric, Japan) for 10min at a distance of 12.5cm. The leaves of the different genotypes were harvested at different time points after the treatment, immediately frozen in liquid nitrogen, and stored at –80 °C until stilbene extraction and RNA analysis.

### Stilbene extraction

To test whether UV-C can induce stilbenes in a stable and reliable manner, leaves of all accessions were collected at the indicated time points: C (control fresh leaf, without UV-C treatment), 0 (just at the end of the 10min UV-C pulse), 3, 6, 24, 48, and 72h, respectively, immediately frozen in liquid nitrogen, and stored at –80 °C until further analysis. The frozen tissue was ground in liquid nitrogen using a pestle and mortar. A 300mg aliquot of fresh weight of powdered leaf tissue was mixed with 1ml of 100% methanol and homogenized for 10min on a platform vortexer in order to maximize uniform sampling and to ensure complete extraction of the stilbenes. The homogenized samples were then centrifuged at 10 000rpm for 10min (Heraeus Biofuge Pico, Osterode, Germany). Before analysis, the supernatant was filtered using a disposable syringe filter (pore size, 0.2 μm; filter-Ø, 15mm; Macherey-Nagel, Düren, Germany). All the experiments were performed under a green safelight (λ_max_ 550nm).

### Stilbene analysis and quantification

For the initial experiments, the stilbenes extracted from *V. rupestris* and *V. quinquangularis* were analysed using high-performance liquid chromatography (HPLC; Agilent 1200 series, Waldbronn, Germany) as described previously (Chang *et al.*, 2011) with minor modifications. To extend the analysis to the numerous cultivars of *V. sylvestris* and *V. vinifera*, liquid chromatography–mass spectrometry (LC-MS) analyses were performed at the metabolomics platform of the Institut National de Recherche Agriculturel (INRA, Université de Strasbourg, Colmar, France) after comparative studies with the same samples had shown that the results between the methods were identical. The analysis method was as follows. Acetonitrile and formic acid of LC-MS grade were supplied by Thermo Fisher (San Jose, CA, USA); water was provided by a Millipore water purification system. Methanolic leaf extracts were analysed using a UHPLC system (Dionex Ultimate 3000, Thermo Fisher Scientific) equipped with a binary pump, an online degasser, a thermostated autosampler, a thermostatically controlled column compartment, and a diode array detector (DAD). Chromatographic separations were performed on a Nucleodur C18 HTec column (50×2mm, 1.8 μm particle size; Macherey-Nagel) maintained at 20 °C. The mobile phase consisted of acetonitrile/formic acid (0.1%, v/v) (eluent A) and water/formic acid (0.1%, v/v) (eluent B) at a flow rate of 0.40ml min^–1^. The gradient elution program was as follows: 0–1min, 85% B; 1–6min, 85% to 5% B; 6–7min, 5% to 85% B; and 7–8min, 85% B. The sample volume injected was 1 μl. The liquid chromatography system was coupled to an Exactive Orbitrap mass spectrometer (Thermo Fischer Scientific) equipped with an electrospray ionization source operating in the negative mode. Parameters were set to 300 °C for ion transfer capillary temperature, and 2500V for needle voltage. Nebulization with nitrogen sheath gas and auxiliary gas was maintained at 50 and 5 arbitrary units, respectively. The spectra were acquired within the *m/z* mass range of 100–1000 atomic mass units (amu), using a resolution of 50 000 at *m/z* 200 amu. The system was calibrated externally using the Thermo Fischer calibration mixture in the range of *m/z* 100–2000 amu, giving a mass accuracy better than 2 ppm. Stilbenes were identified according to their mass spectra, UV absorption spectra, and retention times, and compared with those of authentic standards. The instruments were controlled using the XCalibur software, and data were processed using the XCMS software (Smith *et al.*, 2006). Stilbene quantifications were based on calibration curves obtained with the respective standards. *Trans*-piceid, *trans*-resveratrol, and *trans*-pterostilbene standards were purchased from Sigma-Aldrich (L’Isle d’Abeau, France). (+)-ε-viniferin and (+)-δ-viniferin standards were purchased from Polyphenols Biotech (Villenave d’Ornon, France). *Cis* forms of stilbenes were obtained by photoisomerization under UV light of *trans*-stilbene standard solutions. Solutions containing 0, 5, 10, 25, 50, and 100 μg ^–1^ml of the standards were used for calibrations, with good linearity (*r* ^2^ >0.95). Three independent biological replicates from subsequent seasons were conducted, and all analyses were repeated twice.

### RNA extraction and cDNA synthesis

The leaves of Augster Weiss, Hoe29, Ke53, and Ke83 were harvested at 0, 0.5, 1, 3, 6, and 24h after irradiation, as were those of non-treated controls.

For controlled inoculation with downy mildew (*P. viticola*), a suspension of 40 000 sporangia ml^–1^ was used, as described in detail below, when the screening is described. To circumvent potential modulation of gene expression by a wounding response, this experiment was not conducted with leaf discs, but with entire leaves. The controlled inoculation leaves of Augster Weiss, Hoe29, and Ke83 were harvested at C (control fresh leaf), 120 h-C (control leaf incubated in the absence of *P. viticola* under the same conditions), and 120 h-S (the leaf was infected with *P. viticola* suspension and incubated for 120h), respectively, immediately frozen in liquid nitrogen, and stored at –80 °C until RNA extraction.

Total RNA was isolated using a Spectrum™ Plant Total RNA Kit (Sigma, Deisenhofen) according to the manufacturer’s protocol. The extracted RNA was transcribed into cDNA as described previously (Ismail *et al.*, 2012). The amount of RNA template was 1 μg.

### Semi-quantitative RT–PCR

Semi-quantitative reverse transcription–PCR (RT–PCR) was performed following 30 cycles of 30 s denaturation at 94 °C, 30 s annealing at 60 °C, and 1min synthesis at 68 °C in a conventional PCR cycler (Biometra, Göttingen, Germany) as described previously (Qiao *et al.*, 2010; Chang *et al.*, 2011; Chang and Nick, 2012), using the following primers and the detailed information in Supplementary Table S3 available at *JXB* online: elongation factor-1*α* (*EF1-α*) (sense, 5′–3′ TGTCATGTTGTGTCGTGTCCT; antisense, 5′–3′ CCAAAATATCCGGAGTAAAAGA); phenylalanine ammonium lyase (*PAL*) (sense, 5′–3′ TGCTGACTGGTGAAAAGGTG; antisense, 5′–3′ CGTTCCAAGCACTGAGACAA); resveratrol synthase (*RS*) (sense, 5′–3′ TGGAAGCAACTAGGCATGTG; antisense, 5′–3′ GTGGCTTTTTCCCCCTTTAG); stilbene synthase (*StSy*) (sense, 5′–3′ CCCAATGTGCCCACTTTAAT; antisense, 5’–3’ CTGGGTGAGCAATCCAAAAT); and chalcone synthase (*CHS*) (sense, 5′–3′ GGTGCTCCACAGTGTGTCTACT; antisense, 5′–3′ TACCAACAAGAGAAGGGGAAAA). The PCR was performed with *Taq* polymerase from New England Biolabs (NEB, Frankfurt, Germany) and ThermoPol buffer (NEB). The PCR products were separated as described previously (Ismail *et al.*, 2012).

### Quantitative real-time PCR

Quantitative real-time PCR was performed as described (Svyatyna *et al.*, 2014). To compare the mRNA expression level among different samples, the C_t_ values from each sample were normalized to the value for *EF1-α* as internal standard obtained from the same sample. This internal standard is widely used in studies on stilbenes due to its stability and reliability (Reid *et al.*, 2006; Polesani *et al.*, 2008) and was also found to be very stable in previous work under different biotic and abiotic stress conditions (Qiao *et al.*, 2010; Chang *et al.*, 2011; Chang and Nick, 2012; Ismail *et al.*, 2012). Since actin, which is often used as a housekeeping reference, did not show any deviations from *EF1-α* (Gong and Nick, unpublished), it was decided to calibrate expression data on this internal standard. For each triplicate, these normalized C_t_ values were averaged. The difference between the C_t_ values of the target gene X and those for the *EF1-α* reference R were calculated as follows: ∆C_t_ (X)=C_t_ (X)–C_t_ (R). The final result was expressed as 2^–∆Ct (X)^.

### Principal component analysis and statistical evaluation of metabolomic and genetic data

Principal component analysis (PCA) was performed using the princomp command functioning under R (R Core Team, 2013) using the following argument (cor=T, scores=T). The contribution of the stilbenes to the construction of the axis of the PCA was obtained using R software and the methodology described at http://www.R-project.org/. To infer phylogenetic relationships, DNA was extracted from leaf tissue by a slightly modified cetyltrimethylammonium bromide (CTAB) method (Doyle and Doyle, 1987) using ~25mg of leaf tissue shock-frozen in liquid nitrogen and homogenized. Samples were genotyped at nine microsatellite loci located on different chromosomes (http://www.genres.de/eccdb/vitis/) using the simple sequence repeat (SSR) markers VVS2 (Thomas and Scott, 1993), VVMD07 (Bowers *et al.*, 1996), VVMD25, VVMD27, VVMD28, VVMD32 (Bowers *et al.*, 1999), VrZag62, and VrZag79 (Sefc *et al.*, 1999); the phylogenetic relationship was inferred using the UPGMA method (Sneath and Sokal, 1973) using the software MEGA4 (Tamura *et al.*, 2007) with default settings.

### Screening *V. sylvestris* for susceptibility to downy mildew

To screen differences in the susceptibility of the European wild grape (*V. sylvestris*) accession to downy mildew (*P. viticola*), at least seven leaf discs taken from the fourth to fifth fully expanded leaf of each genotype cultivated in the greenhouse were transferred in a randomized manner to Petri dishes containing 5ml of sterile tap water, inoculated with one droplet of a spore suspension (30 μl for each leaf disc, 40 000 sporangia ml^–1^), which was removed 24h post-inoculation, and incubated in a climate chamber at high humidity and 21 °C (day–night cycle 12 h:12h). Sporulation was first evaluated visually according to Kortekamp (2006) and Genet *et al.* (1997) at 7 days post-inoculation (dpi). In addition, production of spores was scored: each leaf disc was transferred to a 1.5ml tube and complemented with 1ml of 0.1% (v/v) Tween-80 in distilled water. The tube was vigorously shaken (Vortex) to achieve a homogenous suspension, and the concentration of sporangia was determined using a haematocytometer (Fuchs-Rosenthal). The data are means obtained from at least two different years. For all experiments, an isolate was used that is routinely maintained on Müller-Thurgau in the greenhouse of the State Education and Research Center Rheinpfalz.

### Determination of stomatal density

To evaluate stomatal density, glue imprints of fully expanded healthy fresh leaves, harvested from plants grown in the greenhouse of the Botanical Garden of the KIT, were used. Glue imprints were obtained using the lower, abaxial, leaf surfaces of four different leaves of each accession as template. A drop of glue (UHU Hart Modellbaukleber 45510, UHU GmbH & Co. KG, Bühl, Germany) was placed on the respective leaf region near the leaf base. To allow for high-quality imaging, intercostal fields with a sufficiently planar surface were used in the region between the midrib and lateral vein, and covered by a thin and homogenous layer, distributing the glue with the finger tip. After 5–10min, the glue has cured to a thin film, conserving an imprint of the leaf surface. This imprint could then be removed using a pair of tweezers and placed on an object slide in a drop of distilled water. Grey-scale microscopic images were collected from these glue imprints with differential interference contrast (DIC) using a digital imaging system (Zeiss Axio Scope, equipped with a CCD-camera AxioCam). Pictures were recorded at ×20 magnification with 2720×2048 pixels and saved as RGB colour tif files for evaluation with ImageJ. All stomata and epidermal cells on the picture were quantified using the plugin Analyze–Cell Counter. Stomatal density was defined as the ratio of the stomata of one picture per epidermal cells of the same picture, a parameter that was found to be independent of leaf expansion, leaf differentiation, and year (Supplementary Table S4 at *JXB* online). Between 200 and 600 stomata were scored along with epidermal pavement cells to determine the stomatal density. Values represent medians from four independent samples collected over two subsequent vegetation periods.

## Results

### Stilbene accumulation can be triggered by UV-C

**Fig. 1.**
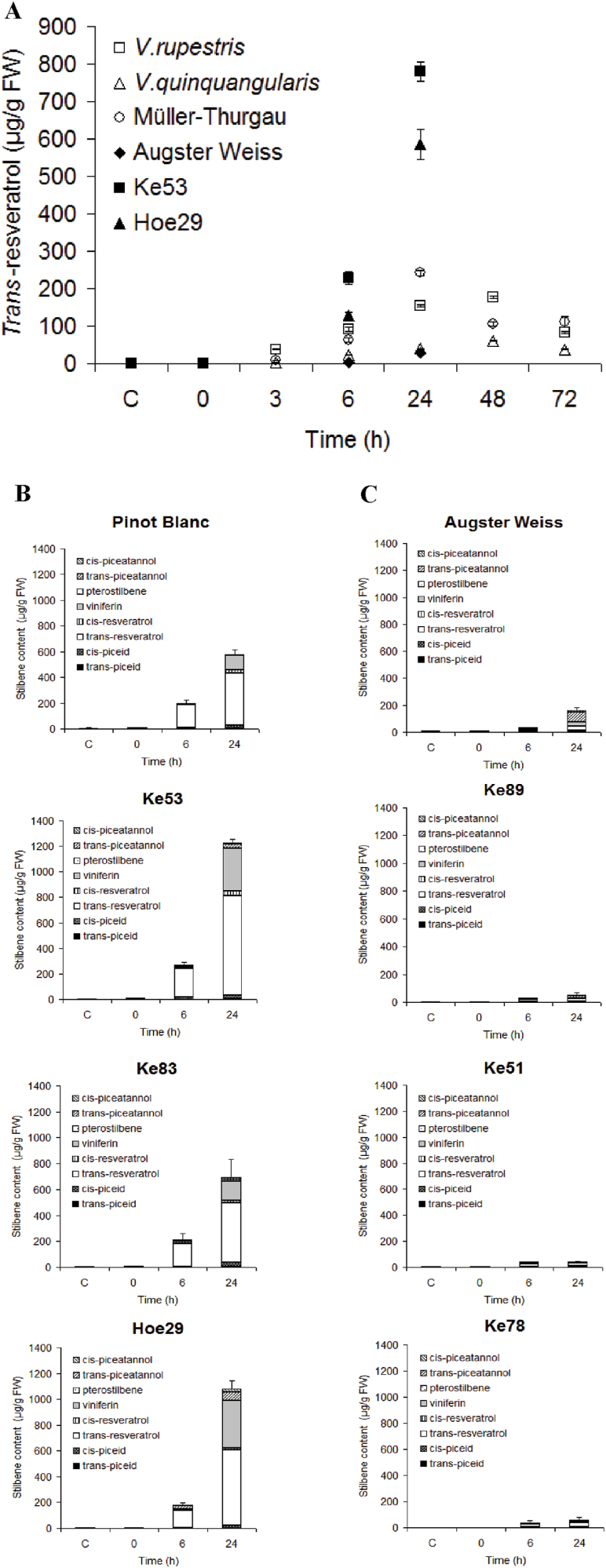
Time courses of stilbene accumulation in different genotypes in response to UV-C. (A) Time courses for the accumulation of *trans*-resveratrol in *V. rupestris*, *V. quinquangularis*, Müller Thurgau, Augster Weiss, Ke53, and Hoe29. Representative time courses for strong stilbene accumulation in Pinot Blanc, Ke53, Ke83, and Hoe29 (B), and weak accumulation in Augster Weiss, Ke89, Ke51, and Ke78 (C). Data represent mean values and standard errors from three independent biological replicates.

In order to compare stilbene inducibility in different genotypes, a trigger is required that is easy to standardize and can be applied to leaf tissue in a reliable manner. From preliminary studies testing different candidate triggers such as methyl jasmonate or inoculation with *P. viticola*, a short pulse of UV-C (10min) was found to produce the most reliable results (Douillet-Breuil *et al.*, 1999). The accumulation of *trans*-resveratrol was first followed over time in response to this UV-C pulse in representative genotypes using HPLC (Fig. 1A). As representative examples, the data are shown for two wild non-*vinifera* species (*V. rupestris*, a North American wild grape, and *V. quinquangularis*, a Chinese wild grape), two *V. vinifera* cultivars (‘Müller Thurgau’, a cultivar commonly grown in the Upper Rhine Region, and ‘Augster Weiss’, a male-sterile ancient variety, which is used for breeding), as well as two *V. sylvestris* genotypes, Hoe29 and Ke53, falling into different subclades of *V. sylvestris*. Prior to the treatment (control), and immediately after the pulse (defined as 0h), the content of *trans*-resveratrol was below the detection limit in all genotypes. The abundance of *trans*-resveratrol increased from 3h after UV-C irradiation, reaching a maximum from 24h to 48h, followed by a decline till 72h. However, the amplitude of the response differed strongly between genotypes, indicating that the accumulation was genotype dependent. For instance, around three times more resveratrol accumulated in *V. rupestris* compared with *V. quinquangularis*, whereas cultivar Müller-Thurgau accumulated more than cultivar Augster Weiss. However, these differences were minor compared with the strong accumulation found in the two *V. sylvestris* genotypes Hoe29 and Ke53. To compare stilbene accumulation between different genotypes, control, 0, 6, and 24h were used as representative time points in the following experiments.

**Fig. 2.**
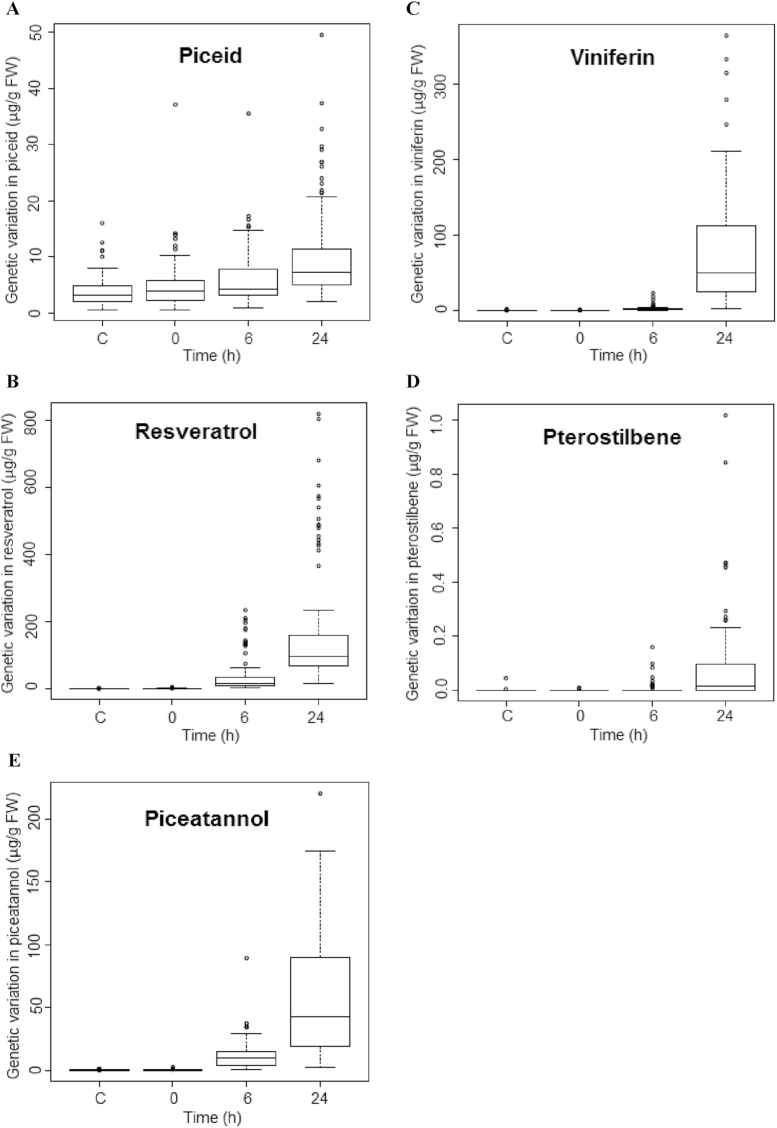
Genetic variation in the stilbene response to UV-C. The accumulation of different stilbene species was determined for 86 genotypes of *V. sylvestris* and a few cultivars. Values are represented in the boxplot format, whereby the box comprises the data for the central 50% of the sample, the horizontal solid line represents the median value, and the dotted line gives the position of the maximal and minimal values excluding the outliers; the outliers are indicated as individual points and were defined as those values that were >1.5 times the upper or lower quartile, respectively. (A) Pooled *cis*- and *trans*-piceid; (B) pooled *cis*- and *trans*-resveratrol; (C) viniferin; (D) pterostilbene; (E) pooled *cis*- and *trans*-piceatannol.

To visualize not only genotypic differences in the total abundance of stilbenes but possibly differences in stilbene profiles, the levels of *trans*-piceid, *cis*-piceid, *trans*-resveratrol, *cis*-resveratrol, ε-viniferin, δ-viniferin, pterostilbene, *trans*-piceatannol, and *cis*-piceatannol were quantified in parallel for the different time points using LC-MS. As shown for a selection of representative genotypes in Fig. 1B and C, there was a large genotypic variation in stilbene inducibility. Whereas UV-C induced a quick and strong accumulation of stilbenes in the genotypes Pinot Blanc, Ke53, Ke83, and Hoe29 (Fig. 1B), the same treatment produced hardly any accumulation in the genotypes Augster Weiss, Ke89, Ke51, and Ke78 (Fig. 1C), even at 24h. Combined analysis of all 86 genotypes (Fig. 2) showed that accumulation of piceid, resveratrol, and piceatannol was observed already 6h after UV-C exposure, whereas viniferins accumulated later and were mostly detected 24h after exposure. This time dependence in the stilbene pattern is shown in Fig. 1B for Pinot Blanc, Ke53, Ke83, and Hoe29. Here, the total stilbene content increased significantly from 6h, which could be mainly attributed to the accumulation of *trans*-resveratrol, whereas at 24h, resveratrol was complemented by viniferins. For example, in Ke53, 234 μg g^–1^ fresh weight (FW) of resveratrol was measured at 6h, with only low levels of viniferin (7 μg g^–1^ FW). In contrast, at 24h, although the content of resveratrol had significantly increased, by >3-fold, to 818 μg g^–1^ FW, during the same time viniferin had increased even more, by >40-fold (333 μg g^–1^ FW). The total stilbene content was therefore 1230 μg g^–1^ FW and exceeded the UV-C-induced stilbene accumulation in genotypes such as Ke89 by >25 times (e.g. even at 24h, the total stilbene content in Ke89 reached only 49 μg g^–1^ FW).

### Genetic variation of stilbene accumulation

In order to evaluate the extent of the genetic variation in defence metabolism present in *V. sylvestris*, stilbene accumulation was followed in 86 genotypes over time in response to UV-C. As shown in Fig. 2A–E, all analysed stilbenes (*cis*- and *trans*-piceid, *cis*- and *trans*-resveratrol, viniferins, pterostilbene, and *cis*- and *trans*-piceatannol) accumulated significantly with increasing time. For piceid, resveratrol, and piceatannol, the increases were observed at early stages (from 6h after UV-C exposure). In contrast, the accumulation of the downstream derivatives viniferins and pterostilbene occurred later: at 6h, these two stilbene species were still not detectable, but had substantially increased at 24h. In all genotypes, resveratrol and viniferins were the predominant stilbenes, and the abundance of viniferins and resveratrol was tightly correlated (the correlations between different types of stilbenes are given in Supplementary Fig. S1 and Supplementary Table S1 at *JXB* online).

In the frame of these general patterns, there was considerable variation as represented by the width of the boxplot bars and the position of the outliers. In some genotypes, such as Pinot Noir, Pinot Blanc, Ke15, Ke20, Ke22, Ke28c, Ke39, Ke53, Ke83, Ke84, Ke95, Ke96, Ke99, Ke103, Hoe17, and Hoe29, much more resveratrol was produced than in the bulk of the populations (Fig. 2B, see the dots on the top of the boxplot at 24h); among those, Ke28c, Ke39, Ke53, Ke84, and Hoe29 also accumulated much more viniferins compared with the bulk of the population (Fig. 2C, see the dots on the top of the boxplot at 24h).

### Two types of stilbene ‘chemovars’

**Fig. 3.**
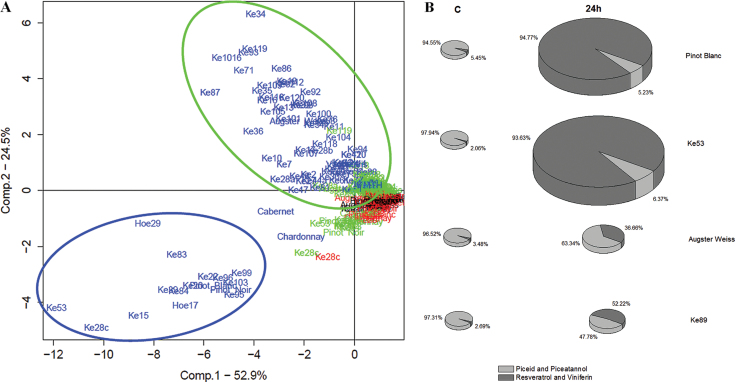
Two stilbene ‘chemovars’ in *V. sylvestris*. (A) Principal component analysis (PCA) over time-dependent accumulation of different stilbene species in a population of 86 genotypes of grapevine. Black, controls (untreated fresh leaf); red (0h), green (6h), and blue (24h) give different time points after a UV-C pulse of 10min. The PCA comprises data from three independent experimental series measuring *cis*-piceid, *trans*-piceid, *cis*-resveratrol, *trans*-resveratrol, viniferin, *cis*-piceatannol, *trans*-piceatannol, and pterostilbene. (B) Representative stilbene profiles of four genotypes. The relative proportion of piceid and piceatannol versus resveratrol and viniferin is shown for the control and 24h after the UV-C pulse. The total abundance of stilbenes is represented by the size of the pie. Pinot Blanc and Ke53 belong to the blue cluster shown in A; Augster Weiss and Ke89 belong to the green cluster.

To understand the factors underlying stilbene variation in *V. sylvestris* (also in relation to some cultivars common in the Upper Rhine Valley and the two non-*vinifera* species from North America and China), the metabolomics data of all 86 genotypes for all time points were subjected to a PCA. As shown in Fig. 3A, the first two principal components could explain 77.4% of the variation between the samples (the contribution of each individual stilbene species to these two principal components is given in Supplementary Table S2 at *JXB* online). Hereby, the amount of stilbenes (Comp. 1) accounted for 52.9% of the variation between the samples, which means that the variation present at 24h could be mainly attributed to the overall content of stilbenes. In contrast to this quantitative trait, Comp. 2 was rather qualitative and based on the composition of the accumulating stilbenes. This explained 24.5% of the variation.

From the PCA at *t*=24h, two clusters of genotypes emerged, which differed in both quantitative and qualitative parameters. The first (smaller) cluster is characterized by the strong ability to accumulate stilbenes, especially in the form of resveratrol and viniferins (Fig. 3A, blue circles). This cluster comprises Pinot Noir, Pinot Blanc, Ke15, Ke20, Ke22, Ke28c, Ke39, Ke53, Ke83, Ke84, Ke95, Ke96, Ke99, Ke103, Hoe17, and Hoe29. The second (larger) cluster comprises genotypes accumulating fewer stilbenes, which a relatively high proportion of piceid and piceatannol.

To illustrate the conclusions from the PCA analysis that the genotypes cluster with respect to the stilbene profile, two representative genotypes arbitrarily selected from each cluster are shown in Fig. 3B. Pinot Blanc and Ke53 belong to the blue (high-stilbene type) cluster, whereas Augster Weiss and Ke89 were chosen from the green (low-stilbene type) cluster. In the controls, the overall abundance of stilbenes was low (represented by the small size of the pie). Those stilbenes that can be detected are almost exclusively present as piceid—the glycosylated form of resveratrol (Fig. 3B). In response to the UV-C pulse, all genotypes accumulated the stilbene species resveratrol and its oxidized form, the viniferins. However, the genotypes from the green (low-stilbene type) cluster (Augster Weiss and Ke89) also accumulated some piceid and piceatannol, which at 24h accounted for ~50–60% of total stilbenes, whereas in genotypes from the blue (high-stilbene type) cluster (Pinot Blanc and Ke53), piceid and piceatannol remained below 7%. When this difference between ‘blue’ and ‘green’ genotypes was tested statistically (Supplementary Figs S2, S3 at *JXB* online), the genotypes from the blue cluster were found to contain significantly more resveratrol and viniferin compared with those from the green cluster. In contrast, the green cluster contained a significantly higher piceatannol/total stilbene ratio.

These data show that there exist two stilbene ‘chemovars’ in *V. sylvestris*. The chemovars of the ‘blue’ cluster accumulate high levels of stilbenes in non-glycosylated form, whereas the chemovars of the ‘green’ cluster accumulate low levels of stilbenes, with a relatively high proportion of piceid and piceatannol.

### Strong stilbene inducibility is distributed in specific clades of *V. sylvestris*

**Fig. 4.**
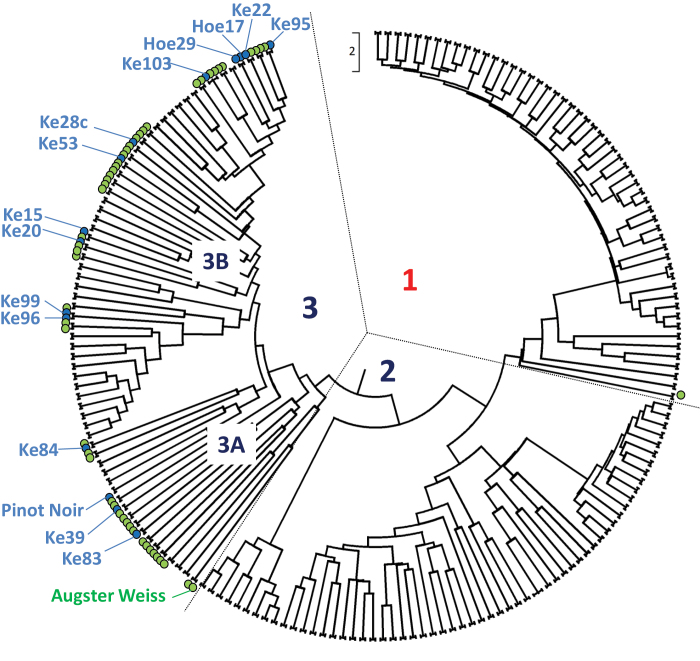
Genetic relationships for stilbene-inducible genotypes of *V. sylvestris*. The incidence of genotypes from the green (piceid-rich chemovars) and the blue (viniferin-rich chemovars) clusters (as defined in Fig. 3A) were plotted into an UPGMA tree over nine SSR markers for 361 taxa of European *V. sylvestris* and *V. vinifera*, and American non-*vinifera*. The tree is drawn to scale, with branch lengths in the same units as those of the evolutionary distances used to infer the phylogenetic tree. 1, non-*inifera* genotypes; 2, *V. sylvestris* genotypes from outside Central Europe; 3, German–Austrian *V. sylvertris*. (This figure is available in colour at *JXB* online.)

The genetic differences in stilbene inducibility represent an interesting genetic resource for resistance breeding. It was therefore decided to determine whether the genotypes of the ‘blue’ (high-stilbene type) cluster (Fig. 3A) are equally distributed over all genotypes from the Ketsch peninsula, or whether they are concentrated on specific clades. The phylogenetic relationship between these genotypes was inferred from microsatellite genotyping and integrated with published data for those microsatellites to comprise a set of 361 taxa of European *V. sylvestris* and *V. vinifera*, and non-European *Vitis* for these nine SSR markers (Fig. 4; Ledesma-Krist *et al.*, 2014). These markers had been selected from the literature, because they are the most informative to resolve relationships in *V. sylvestris*. The topology of the tree was tested by Bayesian clustering, and found to remain very robust after including the first six markers (S. Schröder *et al.*, unpublished results). The accessions from the Ketsch peninsula formed a separate cluster together with *V. sylvestris* from the Upper Danube Valley and *V. vinifera* cultivars current in German vineyards, whereas the *V. sylvestris* accessions from Spain, the Rhône valley, and South East Europe formed a separate cluster, and the non-*vinifera* accessions established a third cluster. When those genotypes that had been analysed with respect to their stilbene inducibility were mapped on this tree, the genotypes of the ‘blue’ (high-stilbene type) cluster were found to be distributed non-homogenously. For instance, among the 15 genotypes where both data sets (SSR markers and stilbene profiles) had been established, only four were found in subcluster 3A, whereas 11 were found in subcluster 3B; within subcluster 3B, five clustered into the right-most branch of the clade.

### Piceid does not serve as a precursor for the biosynthesis of non-glycosylated stilbenes

Some genotypes accumulate relatively high levels of piceid (Fig. 2A, see the dots on the top of the xplots). The glycosylation of piceid protects against oxidation into oxidative dimers, such as viniferins and, therefore, piceid has been proposed to act as the storage form for bioactive resveratrol and viniferins (Regev-Shoshani *et al.*, 2003). It was therefore asked whether piceid might function as a precursor for later release of resveratrol. To illustrate this as an illustration for the UV-C response, the two strong piceid accumulators, Ke28c and Ke10, were selected because these genotypes show comparable resting levels of piceid and resveratrol/viniferin.

**Fig. 5.**
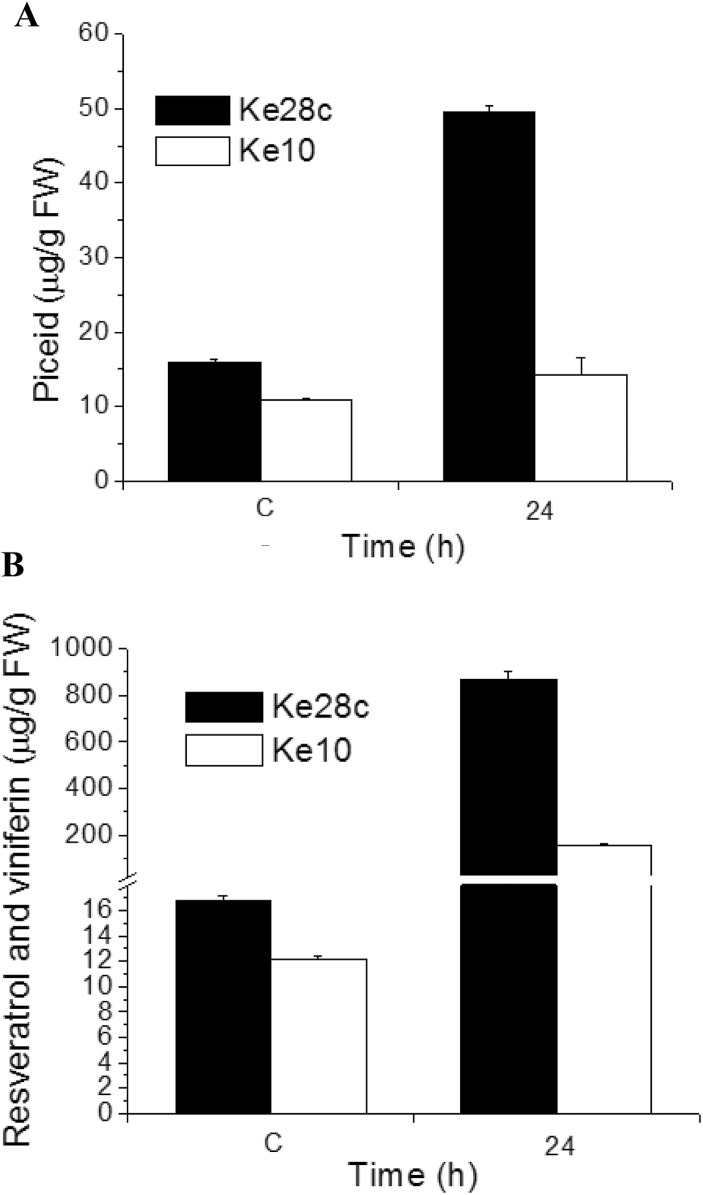
Variation in stilbene inducibility of piceid accumulators. Amounts of piceid (A) and non-glycosylated stilbenes (B) under control conditions and 24h after a UV-C pulse in Ke28c and Ke10. Data represent mean values and standard error from three independent replicates.

Both genotypes showed high basal levels of piceid compared with other genotypes (Fig. 2A). If these high basal levels of piceid were a storage form to produce the bioactive, non-glycosylated, stilbenes, Ke28c and Ke10 should show elevated induction of non-glycosylated stilbenes. However, when they were exposed to UV-C, these two genotypes produced completely different results with respect to stilbene accumulation. Although almost the same amounts of piceid (Fig. 5A) and total stilbenes (Fig. 5B) were measured in the controls, in Ke10, while showing only slightly increased levels of piceid, around >20 times more non-glycosylated stilbenes were induced as compared with the basal level. In contrast, Ke28c accumulated, upon UV-C induction, >3 times the amount of piceid as compared with Ke10, but >6 times the amount of non-glycosylated stilbenes as compared with Ke10.

Therefore, it can be concluded that some of the genotypes with higher basal levels of pre-formed piceid also produce more stilbenes upon induction, but some do not. Even in Ke10, the level of non-glycosylated stilbenes found at 24h exceed the resting level of piceid by >20-fold, which means that the vast majority of induced bioactive stilbenes must be synthesized *de novo* rather than being released from a glycosylated precursor. For the genotypes of the blue cluster, the high levels of resveratrol (Fig. 2B, the dots on the top of the boxplot at 24h) that, in the case of Ke39, Ke53, Ke84, and Hoe29, are accompanied by high amounts of viniferins (Fig. 2C, the dots on the top of the boxplot at 24h) all show only very low resting levels of piceid in control conditions. This means that these genotypes produce their strong induction of stilbenes completely through *de novo* synthesis. Release of resveratrol from pre-formed piceid does not play any role in this induction. To follow the metabolic flow through stilbene formation directly, pulse labelling with radioactive precursors (such as phenylalanine) might be a strategy.

### Response of stilbene-related genes to UV-C

**Fig. 6.**
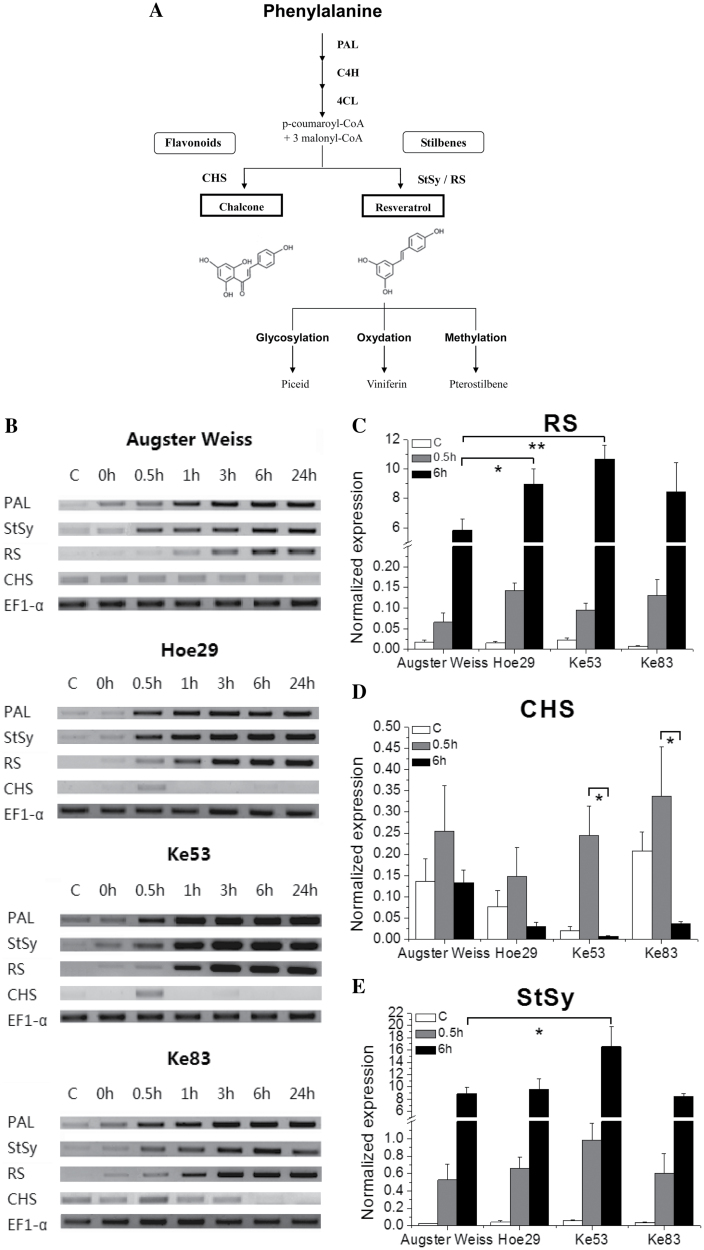
Time courses of the UV-C response for key genes of the phenylpropanoid pathway. (A) Simplified representation of the phenylpropanoid pathway with the positions of phenylalanine ammonium lyase (*PAL*), stilbene synthase (*StSy*), resveratrol synthase (*RS*), and chalcone synthase (*CHS*). (B) Representative agarose gels with the amplificates from semi-quantitative RT–PCR for untreated controls and different time points after irradiation with 10min of UV-C compared with elongation factor *EF1-α* as internal standard. (C–E) Quantification of transcripts by quantitative real-time PCR normalized to the expression of elongation factor *EF1-α*. * and ** indicate differences that are statistically significant at the *P* <0.05 and *P* <0.01 level, respectively. Data represent mean values from five independent experimental series; error bars represent standard errors.

To investigate whether the observed genotypic differences in stilbene accumulation can be correlated with a corresponding transcriptional response, the transcript level of key genes was followed in representative genotypes by semi-quantitative RT–PCR and quantitative real-time PCR. As shown by the simplified stilbene biosynthetic pathway in Fig. 6A, the general activation of the phenylpropanoid pathway was monitored by probing *PAL*, the stilbene branch of the pathway by probing for *StSy* and *RS*, and the competing flavonoid branch via *CHS*. *EF1-α* was used as an internal standard. It should be kept in mind that the stilbene synthase family in grapevine is extremely expanded, with numerous members that are very similar, often even identical in their open reading frames, but differ with respect to their promotors (for reviews, see Parage *et al.*, 2012; Vannozzi *et al.*, 2012). The transcripts picked up by the StSy and RS oligonucleotide primers are therefore likely to stem from different members of this family, and differ partially in their expression patterns (e.g. Qiao *et al.* 2010). In the following, the operational denominators ‘*StSy*’ and ‘*RS*’ will be used. As strong stilbene accumulators, Hoe29, Ke53, and Ke83 were chosen as representative of three different phylogenetic clades of *V. sylvestris* (Fig. 4), whereas Augster Weiss (an ancient cultivar, which is male sterile and therefore often used for molecular breeding) was selected as a representative for the weakly accumulating genotypes.

As shown in Fig. 6B, hardly any transcripts could be detected for the controls and the time point just at the end of the 10min UV-C pulse, irrespective of the genotype, indicating that the basal steady-state levels of these genes are very low. In all strong stilbene accumulators, *PAL* transcripts were found to be induced already 30min after the pulse treatment, whereas in Augster Weiss, the induction of *PAL* transcripts was delayed by 30min and did not reach the same amplitude. The induction of *PAL* transcripts was accompanied by almost simultaneous induction of *StSy* transcripts, whereas *RS* transcripts followed 1–2h later. Again, the response in Augster Weiss was delayed and less pronounced as compared with the strong stilbene accumulators. Interestingly, for Hoe29 and Ke83, the induction of *StSy* did not differ from Augster Weiss, indicating that different stilbene synthase genes can differ in their regulatory pattern (Fig. 6E). Although in these strong stilbene accumulators *PAL* transcripts as well as *StSy* transcripts were induced rapidly, the induction of *CHS* as a key step for the flavonoid pathway remained transient and was shut off between 30min and 60min after the UV-C pulse.

These patterns were then verified by quantitative real-time PCR in the same genotypes. For *RS* transcripts (Fig. 6C), no significant transcript accumulations can be detected under control conditions for any of the tested genotypes. However, already as early as 0.5h, these transcripts had been clearly induced, with the response of Hoe29, Ke53, and Ke83 being stronger than that of Augster Weiss, and this difference had magnified to an almost 2-fold difference at 6h, when the induction in Ke53 is compared with Augster Weiss.

The basal levels for *CHS* transcripts (Fig. 6D) were higher in Augster Weiss and Ke83 compared with Hoe29 and Ke53. Irrespective of this initial difference, transcript levels increased transiently for 0.5h in all genotypes, but this transient increase became significant only in th ecase of Ke53. In all genotypes, the transcript levels had dropped back at 6h, for Hoe29, Ke53, and Ke83 even to a level lower than in the control. In the case of Ke53, the transcripts almost vanished.

The pattern for *StSy* induction (Fig. 6E) resembled that for *RS* transcripts (Fig. 6C), but here the induction was already quite pronounced at 0.5h. Again, the *StSy* transcripts increased more strongly and more rapidly in Ke53 than in Augster Weiss. At 6h, this difference had expanded to a level where the expression of *StSy* in Ke53 was nearly 2-fold that observed in Augster Weiss.

### Expression of *StSy*, *RS*, and *CHS* genes in response to downy mildew

In the previous experiments, genetic differences were found in the inducibility of stilbene that were accompanied by differences in the expression of stilbene synthase genes using UV-C as the trigger. Since the motivation of this work was related to defence, it was important to clarify whether the observed induction by UV-C correlated with an induction by downy mildew. For this purpose, the transcript levels of *StSy*, *RS*, and *CHS* were investigated by quantitative real-time PCR in three representative genotypes: Augster Weiss (a cultivated variety with weak stilbene induction in response to UV), and the two *V. sylvestris* genotypes Hoe29 and Ke83 that showed a strong stilbene response to UV.

**Fig. 7.**
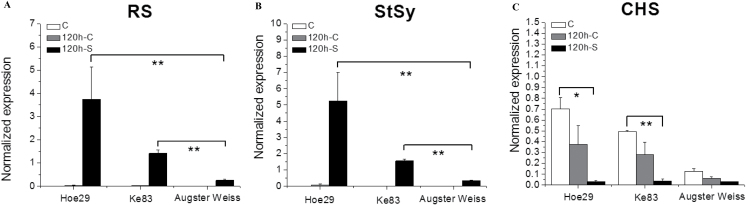
Response of key transcripts of the phenylpropanoid pathway to infection with downy mildew. (A–C) Quantification of transcripts of resveratrol synthase (*RS*), stilbene synthase (*StSy*), and chalcone synthase (*CHS*) by quantitative real-time PCR normalized to the expression of elongation factor *EF1-α*. * and ** indicate differences that are statistically significant at the *P* <0.05 and *P* <0.01 level, respectively. Data represent mean values from three independent experimental series; error bars represent standard errors.

For *RS* transcripts (Fig. 7A), for all three genotypes, no significant transcript accumulation was detected either in the freshly excised leaf (C) or in leaves that had been incubated for 5^d (120 h-C) without inoculation. However, 5 dpi with downy mildew, the expression of *RS* in Hoe29 was strongly induced (by 131-fold compared with the control). This response was >14-fold greater compared with Augster Weiss; in Ke83, this induction was still nearly 6-fold higher than in Augster Weiss.

The pattern of *StSy* (Fig. 7B) was similar to that for *RS* (Fig. 7A); here the expression of *StSy* in Hoe29 was 70-fold greater compared with the control and >15-fold greater compared with Augster Weiss, and in Ke83 was nearly 5-fold greater that observed in Augster Weiss.

In contrast, the abundance of *CHS* transcripts (Fig. 7C), irrespective of the initial difference, decreased compared with the C and 120 h-C for all genotypes. This was most pronounced in Hoe29 and in Ke83, where *CHS* transcripts were more abundant under control conditions. For Augster Weiss, the control levels were lower and thus the decrease was less prominent. Thus, the response *CHS* transcripts represented a mirror image of the situation observed for *RS* and *StSy*.

### Susceptibility to downy mildew is inversely correlated with stilbene inducibility

For the tested representative genotypes, the responses of *RS, StSy*, and *CHS* to inoculation with downy mildew (Fig. 7) correlated with the response of these transcripts to UV-C (Fig. 6). Therefore, a potential correlation between stilbene inducibility by UV-C and the susceptibility to infection by downy mildew in the population was investigated.

*Plasmopara viticola* infects through the stomata, and differences in stomatal density might therefore contribute to variations of infection success. Therefore, the wild *V. vinifera* ssp. *sylvestris* Ketsch population was screened for stomatal density. Preliminary studies had shown that the relative incidence of stomata over the entire population of epidermal cells was a more reliable marker than absolute density (as stomata per area), because this relative value excludes variations caused by differences in cell expansion due to environmental fluctuations (Supplementary Table S4 at *JXB* online). In fact, the values for this relative stomatal density were found to be very stable over two vegetation periods, independent of lighting conditions, and dependent on the genotype.

**Fig. 8.**
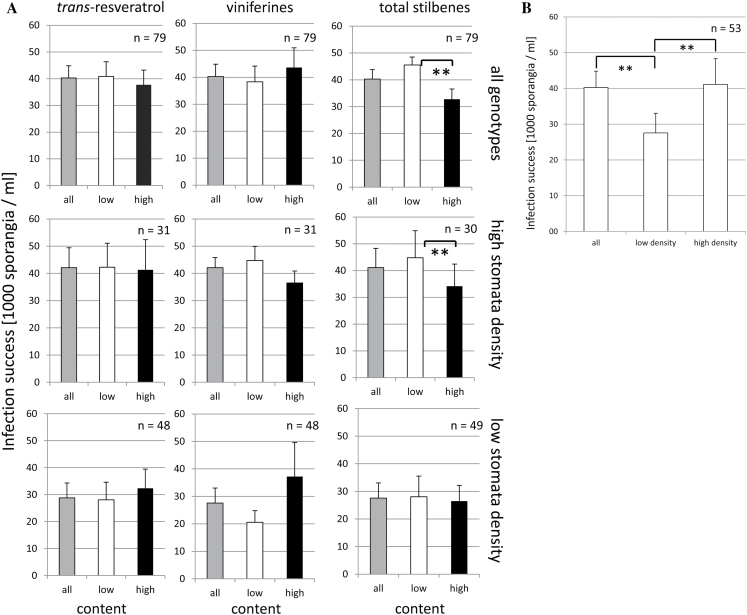
Correlation between UV-induced stilbene accumulation, susceptibility to downy mildew, and stomatal density. (A) Mean susceptibility to downy mildew scored as the concentration of sporangia formed by standardized inoculation in different subsets of the *V. sylvestris* population used in the current study. All, pooled value over the entire population; low, pooled value over those genotypes where the abundance of the respective stilbene was lower than the average of the population; high, pooled value over those genotypes where the abundance was higher than average. Upper row, all genotypes considered; middle row, only genotypes with high stomatal density considered; lower row, only genotypes with low stomatal density considered. (B) The comparison of average infection for all, low (below the median of the population), and high (above the median of the population) densities of stomata (ignoring any difference in the stilbene level). ** indicate differences that are statistically significant atn the *P* <0.01 level. The data represent means and standard errors from three independent biological replicas obtained from at least two different years.

The entire population was now split into a (larger, *n*=59) subset where stilbene contents were lower than average and a (smaller, *n*=20) subset where stilbene contents were higher than average. As a reference, the total abundance of resveratrol and viniferins at 24h after induction was used. When the concentration of sporangia was scored as readout for susceptibility and plotted over these stilbene subsets (Fig. 8A, upper row), there was no significant difference of infections if *trans*-resveratrol and viniferin were considered alone. Since resveratrol can also be oxidized to viniferins non-enzymatically during transport and storage of samples, the correlation of infection with the sum of resveratrol and viniferins was being analysed because this value should be more robust against experimental fluctuations. Here, it was found that the subset of high-stilbene producers had significantly fewer infections compared with the subset of low-stilbene producers. The significance of this finding is at the 99% level.

Since genotypes with a low stomatal density are expected to suffer fewer penetration events, the population was also grouped into two subsets with respect to stomatal density, irrespective of stilbene inducibility (Fig. 8B), and it was found that there was a significantly reduced infection in the group with low stomatal density compared with the average of the entire population and with the high stomatal density group (significance is at the 99% level). No correlation was seen between stomatal density and stilbene inducibility; both traits seemed to be completely uncoupled.

Since the inverse correlation between stilbene levels and infection success was obscured by the fact that genotypes with low stomatal density are less infected even when they perform poorly with respect to stilbene induction, the correlation between infection and stilbene levels was tested separately for those genotypes with high stomatal density (Fig. 8A, middle row) and low stomatal density (Fig. 8A, lower row). Within this subset (Fig. 8A, middle row), the reduction of susceptibility in the high-stilbene producers was even more pronounced.

These data show that both high stilbene inducibility and low stomatal density confer a reduced susceptibility to downy mildew in the *V. sylvestris* population. For high stomatal density, the stilbene content is clearly limiting for infection success, whereas for low stomatal density, the infection success is mostly independent of stilbene content.

## Discussion

Stilbenes, as important phytoalexins, are a central factor for basal immunity of grapevine. In the current study, potential genetic variation in *V. sylvestris*, the ancestor of cultivated grapevine, was probed for with respect to stilbene biosynthetic capacities, for potential use for resistance breeding. Genotypic differences in abundance and profiles of the stilbenes induced in response to a UV-C pulse were shown. Two clusters of genotypes emerged: one cluster with quick and strong accumulation of stilbenes, almost exclusively in the form of the non-glycosylated resveratrol and viniferins, and the second cluster which accumulated fewer stilbenes and a relatively high proportion of piceatannol and the glycosylated piceid. For all 86 genotypes, a time dependence of the stilbene pattern was observed: piceid, resveratrol, and piceatannol accumulated earlier, whereas the viniferins were found later, consistent with a mode of action where resveratrol acts as a precursor for the viniferins. It was further observed that the genotypic differences in stilbene accumulation were preceded by differential accumulation of the transcripts for *PAL*, *StSy*, *RS*, and *CHS*. Taken together, these observations provide evidence for stilbene ‘chemovars’ in *V. sylvestris* (and possibly also in the few vinifera cultivars tested in this study) that differ with respect to the induction of bioactive viniferins correlated with a difference in the inducibility of stilbene synthase.

### On what level is stilbene accumulation controlled?

In the present study, the genotypes from the ‘blue’ (high-stilbene type) cluster (Fig. 3A), such as Pinot Noir, Pinot Blanc, Ke15, Ke20, Ke22, Ke39, Ke53, Ke83, Ke84, Ke95, Ke96, Ke99, Ke103, Hoe17, and Hoe29, accumulate high levels of stilbenes in response to a UV-C pulse (Fig. 2B, C, the dots on the top of the boxplot at 24h), but all show only very low basal levels of stilbenes in control conditions. This means that these genotypes produce their strong induction of stilbenes completely through *de novo* synthesis.

Since stilbenes are derived from the phenylpropanoid pathway (Fig. 6A), the general activation of this pathway was monitored by probing for *PAL*. During evolution, the stilbene branch of the pathway has branched from flavonoid biosynthesis by duplication of the gene encoding *CHS* followed by mutation in the active centre, giving rise to *StSy*/*RS* (Tropf *et al.*, 1994). These enzymes triggering the competing branches of stilbene versus flavonoid biosynthesis are very similar, with only one amino acid difference in the active centre, and the substrate of *StSy*/*RS* is also used by *CHS*, such that both pathways compete for the same precursor. As shown for representative genotypes in Fig. 6B, in all strong stilbene accumulators tested, the induction of *PAL* transcripts was accompanied by an almost simultaneous induction of *StSy* transcripts, whereas *RS* transcripts followed 1–2h later. In contrast, this response was delayed in Augster Weiss and was less pronounced as compared with the strong stilbene accumulators. This indicates that the genotypic differences in the accumulation of stilbenes (Fig. 6) are correlated with the induction of *PAL* transcription as a key regulator of the entire phenylpropanoid pathway. Interestingly, in these strong stilbene accumulators, *CHS*, encoding the key enzyme for the flavonoid pathway, although initially also slightly induced by UV-C, was subsequently down-regulated. This indicates that the phenylpropanoid pathway is, upon activation by UV-C, channelled towards the synthesis of stilbenes, whereas the flavonoid pathway, although initially activated, is rapidly shut down. This might be linked with differential recruitment of *MYB* transcription factors to the *CHS* and *StSy* promotors (Höll *et al.*, 2013).

Although there is a clear correlation between differential activation of *StSy* transcription and the accumulation of stilbenes, it is also clear that the differential induction of *StSy* transcripts (not exceeding a factor of 2–3) cannot account for the much larger differences in the induction of stilbenes (up to a factor of 20). This indicates that transcriptional regulation must be complemented by (still unknown) post-transcriptional mechanisms consistent with findings from elicited grapevine cell lines, where activation of basal immunity by the PAMP flg22 produced a strong accumulation of *StSy* transcripts that was not followed by accumulation of stilbenes (Chang and Nick, 2012). In contrast, the bacterial elicitor Harpin, triggering a cell death-related version of immunity, induced *StSy* transcripts to a similar level, but in addition caused a strong accumulation of stilbenes. An important role for post-transcriptional regulation is also suggested by the fact that a cell culture of Pinot Noir, a genotype belonging to the high-stilbene-type cluster, upon induction of defence preferentially produces the glycosylated piceid (Chang *et al.*, 2011), indicating that epigenetic mechanisms modulate the phenotype.

### Is stilbene inducibility by UV-C a predictor for the response to downy mildew?

To analyse stilbene inducibility on a comparative scale, a pulse of UV-C was used as a reliable and standardized input. However, the motivation for the current study was to explore the potential of *V. sylvestris* as a genetic resource for resistance breeding. This required probing for potential correlations between the UV-C response and the response to a pathogen, such as downy mildew. This correlation is supported by two lines of evidence. (i) The patterns for the induction of stilbene synthesis transcripts (*RS*, *StSy*) along with the competing flavonoid pathway (probed by *CHS*) are highly congruent, irrespective of whether UV-C or inoculation with *P. viticola* are used as the trigger. (ii) Those genotypes that produce high levels of stilbenes in response to UV-C are also found to be significantly less susceptible to infection with downy mildew as compared with those genotypes with low UV inducibility of stilbenes. This correlation becomes even tighter when genotypes with high stomatal density are considered. Thus, the inducibility of stilbene synthesis by a UV-C pulse can be used as a predictor for (partial) resistance to infection with downy mildew.

### Outlook: potential for sustainable viticulture

In grapevine, stilbenes are central to the defence response, with resveratrol in particular effectively preventing pathogen attack (Adrian *et al.*, 1997; Jeandet *et al.*, 2002). Resveratrol is complemented by other metabolic compounds, which harbour efficient antimicrobial activities and are also induced in grapevine as a result of infection or stress (Langcake, 1981; Pezet *et al.*, 2004). Among all stilbenes, oxidized resveratrol oligomers, so-called viniferins, are even more toxic than resveratrol itself and have been shown to inhibit zoospore mobility of *P. viticola*. In contrast, piceid—the glycosylated form of resveratrol—shows no or little toxicity and no antimicrobial activity (Celimene *et al.*, 2001; Pezet *et al.*, 2004). Although stilbenes were induced in all 86 genotypes in response to the UV-C pulse, the genotypes from the blue cluster (Fig. 3A) differed from those of the green cluster not only in accumulating higher levels of stilbenes, but also in producing the non-glycosylated bioactive stilbenes resveratrol and viniferin. The performance of the *V. sylvestris* genotypes after inoculation with different grapevine pathogens such as *P. viticola*, *E. necator*, or *G. bidwellii* are currently being explored and statistically significant correlations have been found between stilbene accumulation and suppression of disease symptoms.

The fact that it is possible to induce stilbene accumulation via an abiotic stress factor (a pulse of UV light) opens up the interesting possibility that immunity might be stimulated by appropriate pre-treatments with abiotic factors. The induction of tolerance to a certain type of stress by a controlled induction of a different stress pathway is termed ‘stress priming’ and has attracted considerable attention in the context of improving agronomical performance under adverse conditions (Beckers and Conrath, 2007). The present study demonstrates that genetic factors enabling strong stilbene inducibility are still present in *V. sylvestris*, and might be reintroduced into cultivated grapes. Since viticulture is not targeted to provide staple food, but a high-quality, high-priced product, quality has clear priority over bulk production. The expected (slight, because inducible) costs for growth and yield expected upon reinstalment of stilbene inducibility would be more than compensated by the reduced costs for chemical plant protection, reduced loss by pathogens, and improved sustainability. Since the ‘blue’ (high-stilbene type) genotypes seem to cluster to specific branches of the phylogenetic tree constructed for the European wild grape, it is also planned to explore the possibility of using the ancestor of cultivated grapevine as a genetic resource for marker-assisted breeding for improved basal immunity.

## Supplementary data

Supplementary data are available at *JXB* online.

Figure S1. Correlations between the amounts of piceid, resveratrol, viniferins, piceatannol, and pterostilbene.

Figure S2. Boxplots of the amounts of each stilbene in the blue (B) and in the green (G) cluster.

Figure S3. Boxplots of the piceatannol/total stilbene ratio in the blue and green cluster.

Table S1. Correlations between the amounts of piceid, resveratrol, viniferins, piceatannol, and pterostilbene.

Table S2. The construction of the stilbenes for each component in principal component analysis.

Table S3. Primer list and literature references used for semi-quantitative RT–PCR and quantitative real-time PCR for this study.

Table S4. Data on developmental and environmental stability of relative stomata incidence.

Supplementary Data
