# Supplementary material for: Genetic diversity of stilbene metabolism in Vitis sylvestris
Source: J Exp Bot. 2015 Apr 6;66(11):3243–57. doi: 10.1093/jxb/erv137 (PMC4449542; doi:10.1093/jxb/erv137)
Supplement: Supplementary Data [file supp_66_11_3243__index.html]

Genetic diversity of stilbene metabolism in Vitis sylvestris — Genetic diversity of stilbene metabolism in Vitis sylvestris — Supplementary Data 

# Genetic diversity of stilbene metabolism in *Vitis sylvestris*

## Supplementary Data

Data files

**Files in this Data Supplement:**

- Supplementary Data - Supplementary Data
