# Supplementary material for: Genetic diversity of stilbene metabolism in Vitis sylvestris
Source: J Exp Bot. 2015 Apr 6;66(11):3243–57. doi: 10.1093/jxb/erv137 (PMC4449542; doi:10.1093/jxb/erv137)
Supplement: Supplementary Data [file supp_erv137_jexbot141622_file001.pdf]

Genetic diversity of stilbene metabolism in *Vitis sylvestris*

Dong Duan, David Halter, Raymonde Baltenweck, Christine Tisch, Viktoria Tröster, Andreas Kortekamp, Philippe Hugueney and Peter Nick

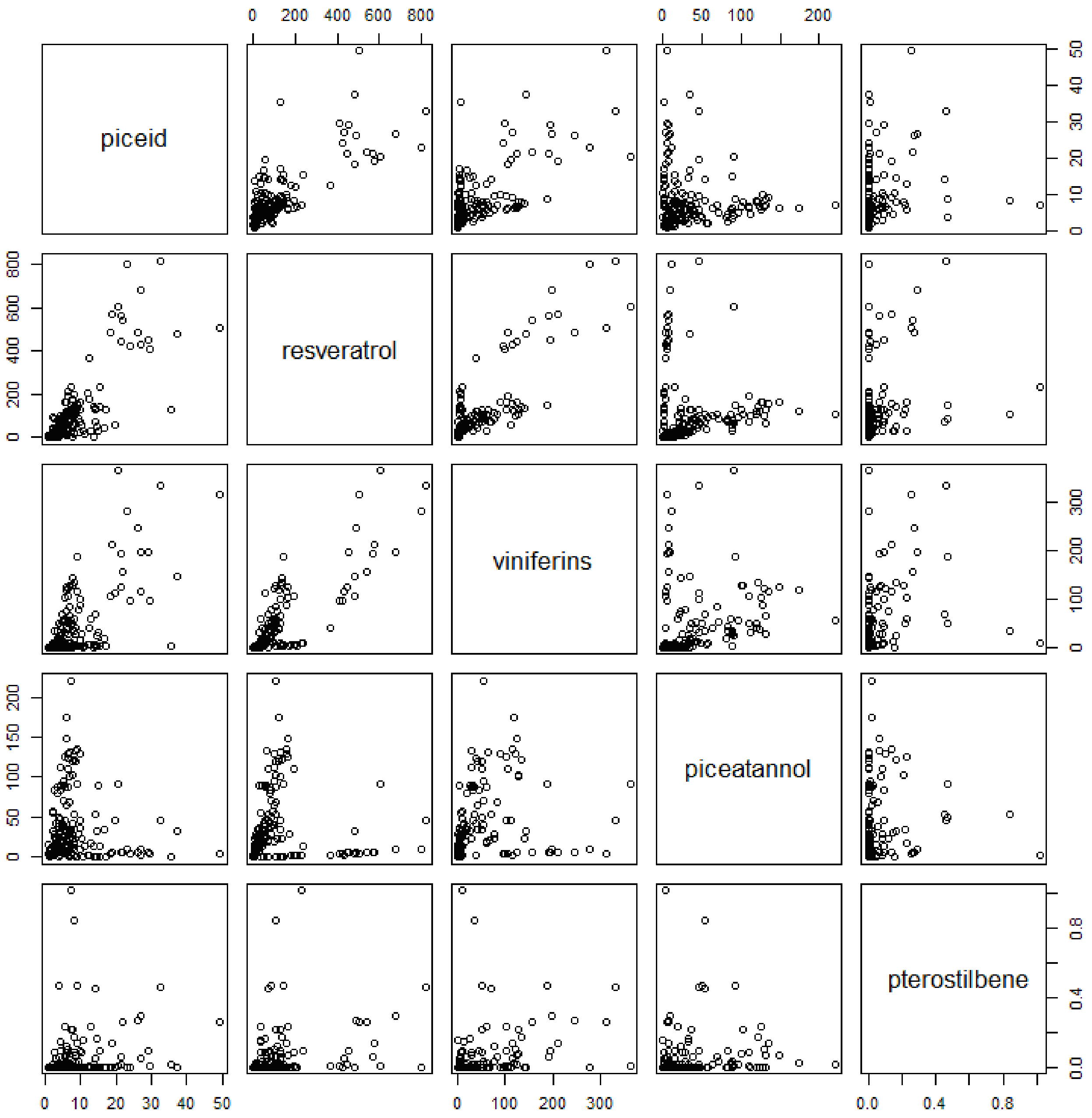

Figure S1. Correlations between the amounts of piceid, resveratrol, viniferins, piceatannol and pterostilbene.

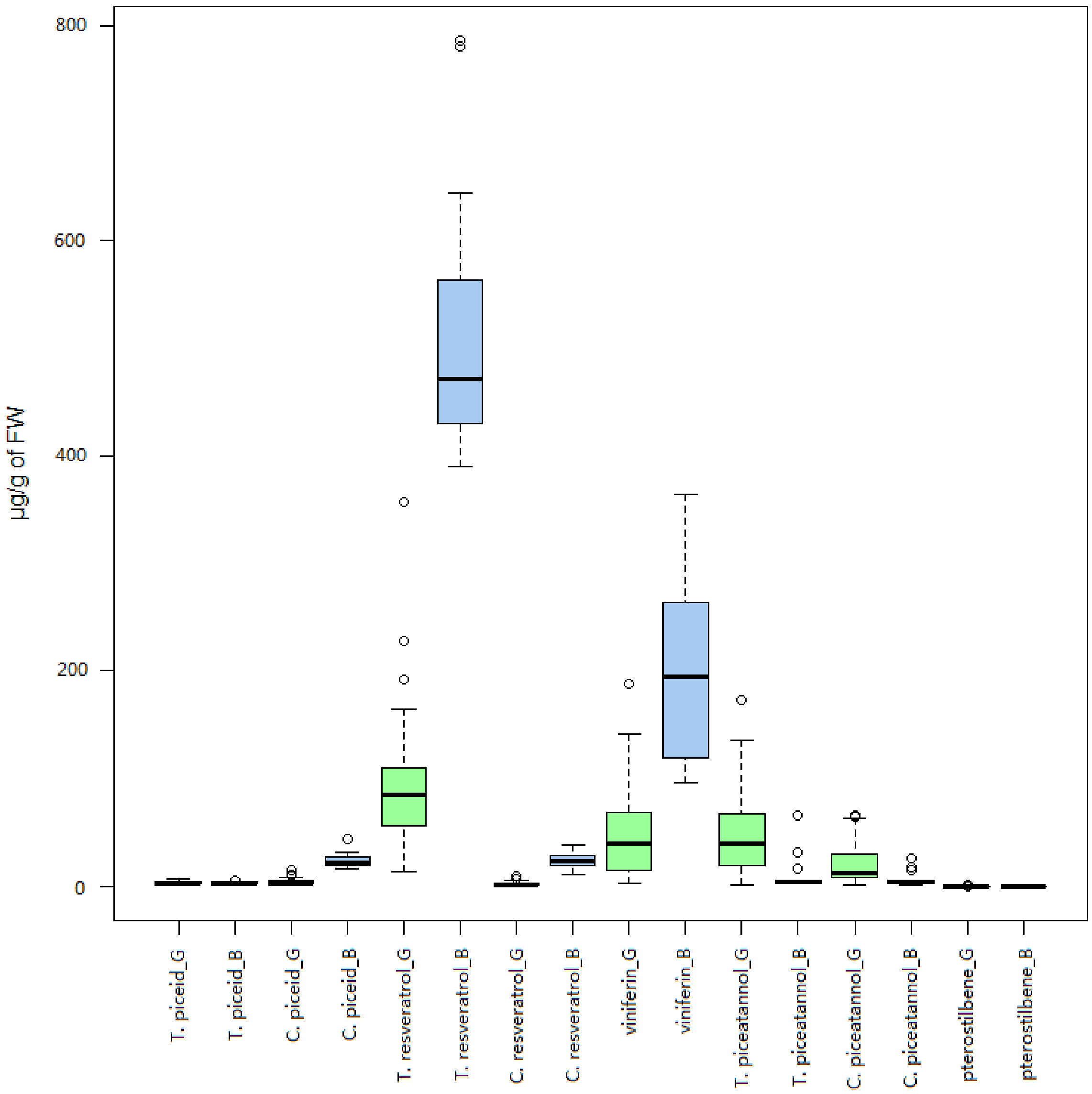

Figure S2. Boxplots of the amounts of each stilbene in the blue (B) and in the green (G) cluster. The amounts of trans-piceid and pterostilbene are not statistically different in both clusters (t-test, *p*-val=0.6951 and 0.006 respectively), whereas, cis-piceid, trans-resveratrol, cis-resveratrol, viniferins, trans-piceatannol, cis-piceatannol are statistically differently accumulated in the blue cluster in comparison to the green one (t-test, *p*-val=5.51 x 10^{-9}, 3.1 x 10^{-10}, 1.77 x 10^{-9}, 5.37 x 10^{-6}, 4.71 x 10^{-8} and 7.48 x 10^{-7}, respectively).

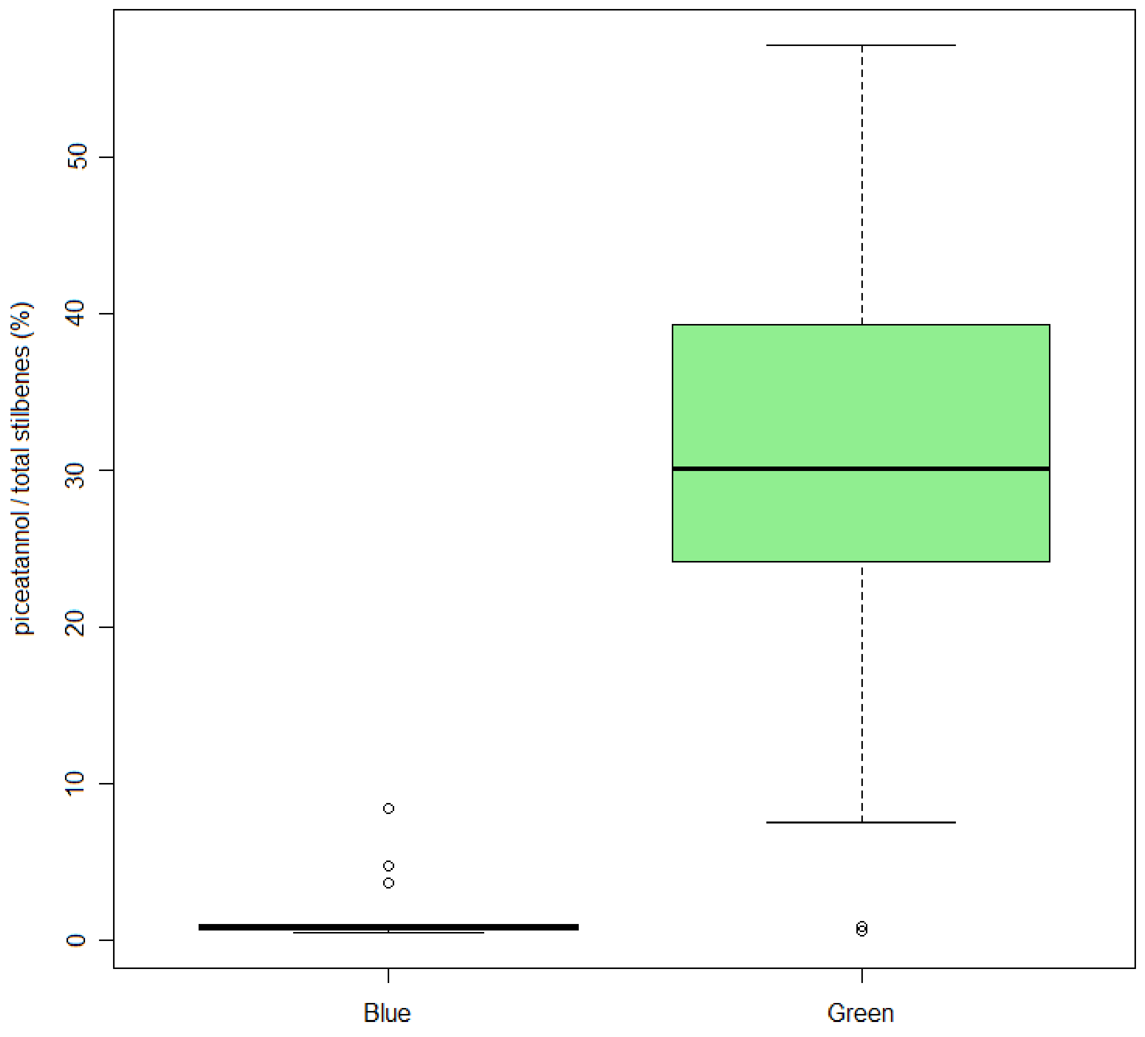

Figure S3. Boxplots of the piceatannol/total stilbene ratio in the blue and green cluster. The ratio is statistically greater in the green cluster in comparison to the blue one (t-test, *p*-val<2.2 x 10^{-16}).

Table S1. Correlations between the amounts of piceid, resveratrol, viniferins, piceatannol and pterostilbene.

	piceid	resveratrol	viniferins	piceatannol	pterostilbene
piceid	1.00	0.81	0.74	-0.36	0.16
resveratrol	0.81	1.00	0.83	-0.31	0.18
viniferins	0.74	0.83	1.00	-0.05	0.20
piceatannol	-0.37	-0.31	-0.05	1.00	-0.11
pterostilbene	0.16	0.18	0.20	-0.11	1.00

Table S2. The construction of the stilbenes for each component in principal component analysis.

	Comp.1	Comp.2
trans-piceid	-0.395	0.282
cis-piceid	-0.323	-0.395
trans-resveratrol	-0.433	-0.231
cis-resveratrol	-0.401	-0.339
viniferin	-0.449	
trans-piceatannol	-0.239	0.533
cis-piceatannol	-0.265	0.533
pterostilbene	-0.251	

Table S3. Primers list and literature references used for semi-quantitative RT-PCR and quantitative real-time PCR for this study.

**Name**	**GeneBank accession no.**	**Primer sequence 5'-3'**	**Reference**
EF1-α	EC959059	Sense: 5'- -3' TGTCATGTTGTGTCGTGTCCT<br>Antisense: 5'- -3' CCAAAATATCCGGAGTAAAAGA	This paper
PAL	X75967	Sense: 5'- -3' TGCTGACTGGTGAAAAGGTG<br>Antisense: 5'- -3' CGTTCCAAGCACTGAGACAA	Belhadj *et al.* (2008)
RS	AF274281	Sense: 5'- -3' TGGAAGCAACTAGGCATGTG<br>Antisense: 5'- -3' GTGGCTTTTTCCCCCTTTAG	This paper
StSy	X76892	Sense: 5'- -3' CCCAATGTGCCCACTTTAAT<br>Antisense: 5'- -3' CTGGGTGAGCAATCCAAAAT	This paper
CHS	AB066274	Sense: 5'- -3' GGTGCTCCACAGTGTGTCTACT<br>Antisense: 5'- -3' TACCAACAAGAGAAGGGGAAAA	Belhadj *et al.* (2008)

Table S4. Stomatal density (as frequency of guard cell pairs per total number of epidermal cells) is independent of leaf expansion, leaf differentiation, and year. n indicates the number of leaves collected from different plants. From each leaf between 200-600 stomata were scored to determine stomatal density.

**Genotype**	**parameter**	**Stomatal density** [fraction of guard cells]
Ke110	Leaf expansion	
n = 8	Just emerged	0.092 ± 0.006
n = 4	During expansion	0.089 ± 0.005
n = 16	Fully expanded	0.092 ± 0.004
Ke83	Leaf differentiation	
n = 8	Small, green	0.060 ± 0.004
n = 4	Medium size, green	0.063 ± 0.003
n = 4	Large, green	0.062 ± 0.011
n = 16	Large, anthocyanin	0.065 ± 0.002
V. rupestris	Year	
n = 4	Year 1	0.099 ± 0.005
n = 16	Year 2	0.102 ± 0.003
